# Crystal structure of bis­[2-(benzo­thia­zol-2-yl)phenolato-κ^2^ *N*,*O*]copper(II)

**DOI:** 10.1107/S2056989015015303

**Published:** 2015-08-22

**Authors:** Namhun Kim, Sung Kwon Kang

**Affiliations:** a Department of Chemistry, Chungnam National University, Daejeon 305-764, Republic of Korea

**Keywords:** crystal structure, Cu(II) complex, benzo­thia­zolphenol, hydrogen bonding, π–π inter­actions

## Abstract

In the title complex, [Cu(C_13_H_8_NOS)_2_], the Cu^II^ atom is coordinated by two N atoms and two O atoms from two bidentate benzo­thia­zolphenolate ligands, forming a distorted tetra­hedral geometry [dihedral angle between two N—Cu—O planes: 45.1 (2)°]. The dihedral angles between the benzo­thia­zole ring systems and the phenol rings are 4.1 (4) and 5.8 (4)°, indicating an almost planar geometry. Weak intra- and inter­molecular C—H⋯O hydrogen bonds are observed. In the crystal, weak π–π inter­actions between aromatic and thia­zole rings [centroid–centroid distances = 3.626 (3) and 3.873 (3) Å] link the mol­ecules into a two-dimensional supra­molecular network along the *bc* plane.

## Related literature   

For background to benzo­thia­zole complexes and their applications, see: López-Banet *et al.* (2014 ▸); Liu *et al.* (2011 ▸); Booysen *et al.* (2010 ▸); Henary & Fahrni (2002 ▸). For the structures and luminescent properties of metal complexes, see: Yu *et al.* (2003 ▸); Katkova *et al.* (2011 ▸); Balashova *et al.* (2013 ▸); Wang *et al.* (2002 ▸).
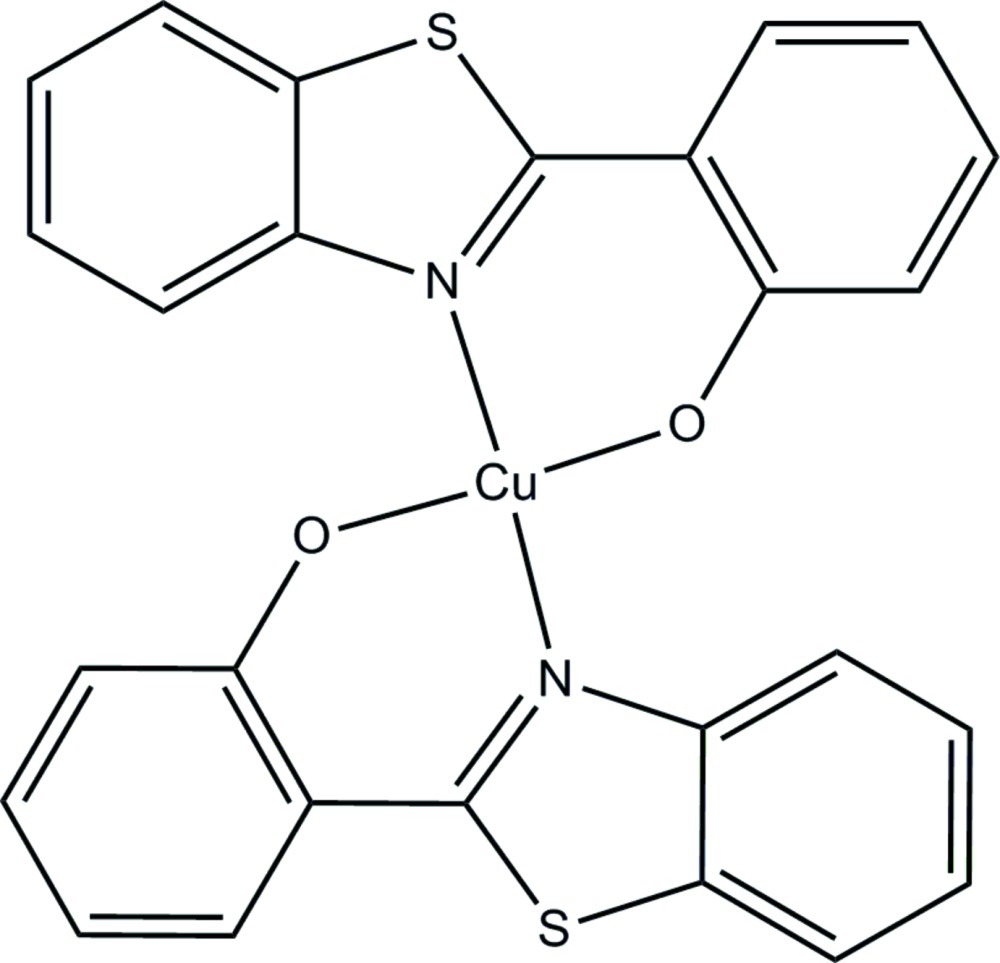


## Experimental   

### Crystal data  

[Cu(C_13_H_8_NOS)_2_]
*M* *_r_* = 516.07
Monoclinic, 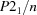

*a* = 7.8177 (17) Å
*b* = 21.195 (5) Å
*c* = 12.495 (3) Å
β = 91.077 (2)°
*V* = 2070.1 (8) Å^3^
*Z* = 4
Mo *K*α radiation
μ = 1.29 mm^−1^
*T* = 296 K
0.08 × 0.06 × 0.05 mm

### Data collection  

Bruker SMART CCD area-detector diffractometer
Absorption correction: multi-scan (*SADABS*; Bruker, 2002 ▸) *T*_min_ = 0.902, *T*_max_ = 0.925
21140 measured reflections
3855 independent reflections
2045 reflections with *I* > 2σ(*I*)
*R*_int_ = 0.149

### Refinement  

*R*[*F* ^2^ > 2σ(*F* ^2^)] = 0.086
*wR*(*F* ^2^) = 0.224
*S* = 1.08
3855 reflections
298 parameters
H-atom parameters constrained
Δρ_max_ = 0.98 e Å^−3^
Δρ_min_ = −1.21 e Å^−3^

### 

Data collection: *SMART* (Bruker, 2002 ▸); cell refinement: *SAINT* (Bruker, 2002 ▸); data reduction: *SAINT*; program(s) used to solve structure: *SHELXS2013* (Sheldrick, 2008 ▸); program(s) used to refine structure: *SHELXL2013* (Sheldrick, 2015 ▸); molecular graphics: *ORTEP-3 for Windows* (Farrugia, 2012 ▸); software used to prepare material for publication: *publCIF* (Westrip, 2010 ▸).

## Supplementary Material

Crystal structure: contains datablock(s) I, New_Global_Publ_Block. DOI: 10.1107/S2056989015015303/bq2400sup1.cif

Structure factors: contains datablock(s) I. DOI: 10.1107/S2056989015015303/bq2400Isup2.hkl

**Fig. 1.**
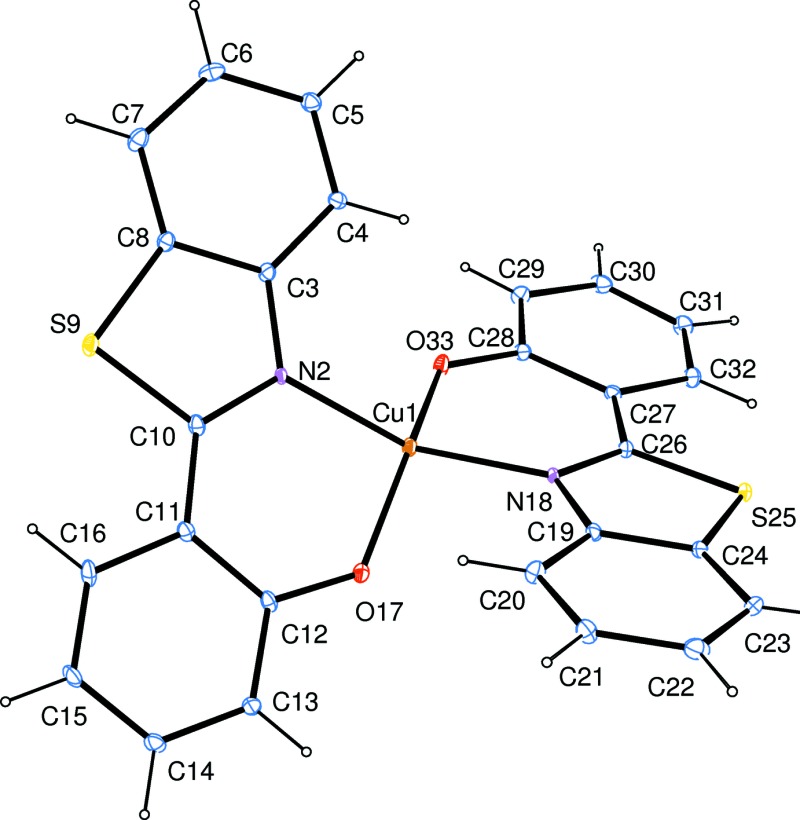
Molecular structure of the title complex, showing the atom-numbering scheme and 30% probability ellipsoids.

**Fig. 2.**
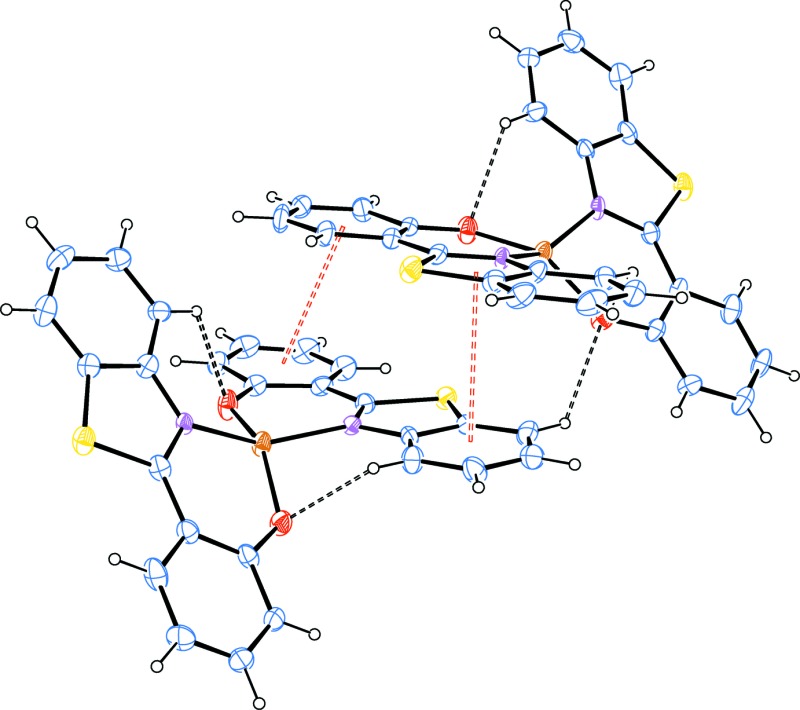
Dimeric formation via C—H···O (black dashed lines) and π-π (red) interactions.

. DOI: 10.1107/S2056989015015303/bq2400fig1.tif
Mol­ecular structure of the title complex, showing the atom-numbering scheme and 30% probability ellipsoids.

via . DOI: 10.1107/S2056989015015303/bq2400fig2.tif
Dimeric formation *via* C—H⋯O (black dashed lines) and π-π (red) inter­actions.

**Fig. 3.**
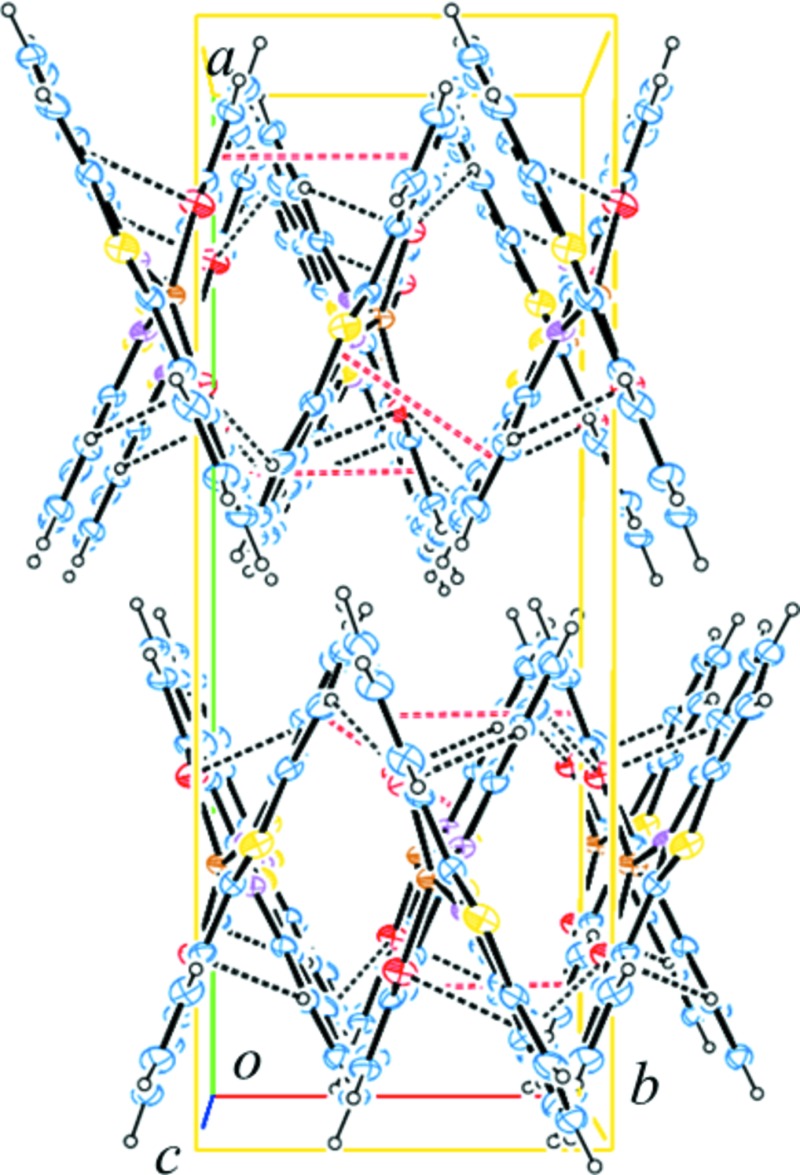
Part of the crystal structure of the title complex, showing the 2-D network of molecules linked by intermolecular C—H···O hydrogen bonds (black dashed lines) and π-π interactions (red).

. DOI: 10.1107/S2056989015015303/bq2400fig3.tif
Part of the crystal structure of the title complex, showing the 2-D network of mol­ecules linked by inter­molecular C—H⋯O hydrogen bonds (black dashed lines) and π-π inter­actions (red).

CCDC reference: 1419096

Additional supporting information:  crystallographic information; 3D view; checkCIF report

## Figures and Tables

**Table 1:** Hydrogen-bond geometry (, )

*D*H*A*	*D*H	H*A*	*D* *A*	*D*H*A*
C4H4O33	0.93	2.41	2.997(12)	121
C7H7O17^i^	0.93	2.59	3.305(13)	134
C20H20O17	0.93	2.42	3.000(13)	121
C23H23O33^ii^	0.93	2.61	3.303(13)	132

Symmetry codes: (i) 
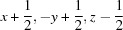
; (ii) 
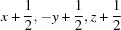
.

## supplementary crystallographic information

### S1.1. Synthesis and crystallization

To a solution of 2-(2-hy­droxy­phenyl)­benzo­thia­zole (0.227 g, 1.0 mmol) in EtOH (15 ml) was added a 1N NaOH solution slowly until pH = 8 at room temperature. After 6 h of stirring, a solution of Cu(NO_3_)_2_.3H_2_O (0.121g, 0.50 mmol) in EtOH (15 ml) was added. After 24 h of stirring at room temperature, the product was isolated as a dark green powder by removing the solvent. Green single crystals of the title complex were obtained by slow evaporation of its concentrated solution in di­chloro­methane at room temperature.

### S1.2. Refinement

All H atoms were positioned geometrically and refined using a riding model, with C—H = 0.93 Å, and with U_iso_(H) = 1.2U_eq_(C).

### Crystal data

**Table:**

[Cu(C_13_H_8_NOS)_2_]	*F*(000) = 1052
*M**_r_* = 516.07	*D*_x_ = 1.656 Mg m^−^^3^
Monoclinic, *P*2_1_/*n*	Mo *K*α radiation, λ = 0.71073 Å
*a* = 7.8177 (17) Å	Cell parameters from 1300 reflections
*b* = 21.195 (5) Å	θ = 3.1–18.7°
*c* = 12.495 (3) Å	µ = 1.29 mm^−^^1^
β = 91.077 (2)°	*T* = 296 K
*V* = 2070.1 (8) Å^3^	Block, green
*Z* = 4	0.08 × 0.06 × 0.05 mm

### Data collection

**Table:**

Bruker SMART CCD area-detector diffractometer	2045 reflections with *I* > 2σ(*I*)
Radiation source: fine-focus sealed tube	*R*_int_ = 0.149
φ and ω scans	θ_max_ = 25.5°, θ_min_ = 1.9°
Absorption correction: multi-scan (*SADABS*; Bruker, 2002)	*h* = −9→9
*T*_min_ = 0.902, *T*_max_ = 0.925	*k* = −25→25
21140 measured reflections	*l* = −15→15
3855 independent reflections	

### Refinement

**Table:**

Refinement on *F*^2^	0 restraints
Least-squares matrix: full	Hydrogen site location: inferred from neighbouring sites
*R*[*F*^2^ > 2σ(*F*^2^)] = 0.086	H-atom parameters constrained
*wR*(*F*^2^) = 0.224	*w* = 1/[σ^2^(*F*_o_^2^) + (0.0651*P*)^2^ + 14.3316*P*] where *P* = (*F*_o_^2^ + 2*F*_c_^2^)/3
*S* = 1.08	(Δ/σ)_max_ = 0.002
3855 reflections	Δρ_max_ = 0.98 e Å^−^^3^
298 parameters	Δρ_min_ = −1.21 e Å^−^^3^

### Special details

**Table:**

Geometry. All e.s.d.'s (except the e.s.d. in the dihedral angle between two l.s. planes) are estimated using the full covariance matrix. The cell e.s.d.'s are taken into account individually in the estimation of e.s.d.'s in distances, angles and torsion angles; correlations between e.s.d.'s in cell parameters are only used when they are defined by crystal symmetry. An approximate (isotropic) treatment of cell e.s.d.'s is used for estimating e.s.d.'s involving l.s. planes.

### Fractional atomic coordinates and isotropic or equivalent isotropic displacement parameters (Å^2^)

**Table:**

	*x*	*y*	*z*	*U*_iso_*/*U*_eq_	
Cu1	0.05754 (17)	0.25058 (6)	0.87875 (8)	0.0412 (4)	
N2	0.1275 (10)	0.2275 (3)	0.7303 (5)	0.0320 (18)	
C3	0.2057 (13)	0.1712 (4)	0.7036 (7)	0.040 (2)	
C4	0.2656 (13)	0.1241 (5)	0.7711 (8)	0.042 (2)	
H4	0.2553	0.1287	0.8447	0.050*	
C5	0.3393 (14)	0.0711 (5)	0.7315 (9)	0.050 (3)	
H5	0.3784	0.0397	0.7778	0.060*	
C6	0.3563 (15)	0.0639 (5)	0.6218 (10)	0.057 (3)	
H6	0.4056	0.0272	0.5955	0.069*	
C7	0.3018 (16)	0.1096 (5)	0.5514 (9)	0.056 (3)	
H7	0.3172	0.1052	0.4782	0.068*	
C8	0.2228 (13)	0.1630 (5)	0.5931 (7)	0.042 (3)	
S9	0.1445 (4)	0.22811 (13)	0.52567 (19)	0.0492 (7)	
C10	0.0888 (13)	0.2633 (4)	0.6462 (7)	0.041 (3)	
C11	0.0162 (13)	0.3243 (5)	0.6463 (7)	0.042 (2)	
C12	−0.0326 (13)	0.3561 (4)	0.7415 (7)	0.041 (2)	
C13	−0.0964 (14)	0.4177 (5)	0.7335 (8)	0.048 (3)	
H13	−0.1232	0.4395	0.7955	0.058*	
C14	−0.1203 (15)	0.4465 (5)	0.6363 (9)	0.051 (3)	
H14	−0.1662	0.4870	0.6329	0.062*	
C15	−0.0770 (16)	0.4161 (5)	0.5440 (9)	0.058 (3)	
H15	−0.0936	0.4357	0.4780	0.070*	
C16	−0.0096 (16)	0.3572 (5)	0.5493 (8)	0.057 (3)	
H16	0.0210	0.3376	0.4860	0.069*	
O17	−0.0164 (10)	0.3307 (3)	0.8372 (5)	0.0496 (19)	
N18	0.1264 (10)	0.2750 (3)	1.0266 (5)	0.0340 (19)	
C19	0.2058 (13)	0.3322 (4)	1.0571 (7)	0.040 (2)	
C20	0.2587 (14)	0.3789 (5)	0.9866 (8)	0.048 (3)	
H20	0.2450	0.3738	0.9131	0.057*	
C21	0.3307 (15)	0.4321 (5)	1.0281 (9)	0.054 (3)	
H21	0.3648	0.4640	0.9819	0.064*	
C22	0.3545 (15)	0.4401 (6)	1.1372 (11)	0.066 (4)	
H22	0.4041	0.4771	1.1631	0.079*	
C23	0.3056 (16)	0.3940 (5)	1.2079 (9)	0.056 (3)	
H23	0.3249	0.3988	1.2811	0.068*	
C24	0.2262 (13)	0.3397 (5)	1.1673 (7)	0.044 (3)	
S25	0.1515 (4)	0.27492 (13)	1.23252 (18)	0.0478 (7)	
C26	0.0923 (12)	0.2389 (4)	1.1125 (7)	0.038 (2)	
C27	0.0203 (13)	0.1783 (4)	1.1113 (7)	0.039 (2)	
C28	−0.0212 (13)	0.1443 (5)	1.0149 (7)	0.039 (2)	
C29	−0.0817 (13)	0.0823 (5)	1.0220 (8)	0.047 (3)	
H29	−0.1032	0.0591	0.9600	0.056*	
C30	−0.1096 (16)	0.0555 (5)	1.1211 (9)	0.059 (3)	
H30	−0.1487	0.0142	1.1251	0.071*	
C31	−0.0805 (16)	0.0889 (5)	1.2129 (9)	0.059 (3)	
H31	−0.1069	0.0712	1.2786	0.070*	
C32	−0.0128 (15)	0.1482 (5)	1.2087 (8)	0.057 (3)	
H32	0.0121	0.1692	1.2724	0.068*	
O33	−0.0039 (10)	0.1691 (3)	0.9204 (5)	0.053 (2)	

### Atomic displacement parameters (Å^2^)

**Table:**

	*U*^11^	*U*^22^	*U*^33^	*U*^12^	*U*^13^	*U*^23^
Cu1	0.0658 (9)	0.0340 (6)	0.0238 (5)	−0.0022 (7)	0.0006 (5)	0.0018 (5)
N2	0.049 (5)	0.028 (4)	0.019 (3)	0.000 (4)	−0.005 (3)	0.003 (3)
C3	0.049 (7)	0.038 (6)	0.032 (5)	−0.004 (5)	0.009 (5)	0.006 (4)
C4	0.039 (6)	0.043 (6)	0.043 (6)	0.005 (5)	−0.001 (5)	0.001 (5)
C5	0.052 (8)	0.047 (7)	0.051 (7)	0.002 (6)	0.004 (6)	0.008 (5)
C6	0.057 (9)	0.037 (6)	0.078 (9)	−0.001 (5)	0.019 (7)	−0.010 (6)
C7	0.075 (9)	0.045 (7)	0.049 (7)	−0.013 (6)	0.011 (6)	−0.008 (5)
C8	0.045 (7)	0.044 (6)	0.038 (5)	−0.005 (5)	0.006 (5)	0.001 (4)
S9	0.069 (2)	0.0508 (16)	0.0282 (12)	−0.0052 (14)	0.0067 (12)	0.0016 (11)
C10	0.049 (6)	0.039 (7)	0.034 (5)	−0.011 (5)	−0.001 (4)	0.008 (4)
C11	0.047 (7)	0.040 (6)	0.038 (5)	−0.007 (5)	−0.005 (5)	0.006 (4)
C12	0.049 (7)	0.036 (6)	0.037 (5)	−0.012 (5)	−0.013 (5)	0.010 (4)
C13	0.064 (8)	0.034 (6)	0.045 (6)	0.004 (5)	−0.012 (5)	−0.005 (5)
C14	0.064 (8)	0.030 (6)	0.059 (7)	−0.002 (5)	−0.010 (6)	0.005 (5)
C15	0.079 (9)	0.046 (7)	0.049 (7)	0.005 (6)	−0.010 (6)	0.020 (5)
C16	0.081 (9)	0.061 (8)	0.030 (5)	−0.019 (7)	−0.007 (5)	0.012 (5)
O17	0.073 (5)	0.045 (4)	0.030 (4)	0.007 (4)	−0.001 (3)	−0.001 (3)
N18	0.054 (5)	0.029 (4)	0.019 (3)	0.009 (4)	−0.003 (3)	0.004 (3)
C19	0.048 (7)	0.033 (5)	0.037 (5)	0.003 (5)	−0.018 (5)	−0.003 (4)
C20	0.054 (7)	0.045 (6)	0.044 (6)	−0.015 (5)	0.004 (5)	−0.002 (5)
C21	0.055 (8)	0.049 (7)	0.057 (7)	−0.013 (6)	−0.014 (6)	0.007 (5)
C22	0.059 (9)	0.045 (7)	0.093 (10)	0.001 (6)	−0.025 (8)	−0.014 (7)
C23	0.075 (9)	0.051 (7)	0.043 (6)	0.015 (6)	−0.007 (6)	−0.015 (5)
C24	0.051 (7)	0.039 (6)	0.041 (6)	0.011 (5)	−0.016 (5)	−0.010 (4)
S25	0.070 (2)	0.0490 (15)	0.0244 (12)	0.0134 (14)	−0.0030 (12)	−0.0009 (11)
C26	0.042 (6)	0.044 (7)	0.027 (5)	0.006 (5)	0.001 (4)	0.000 (4)
C27	0.049 (7)	0.031 (5)	0.038 (5)	0.004 (5)	0.005 (5)	0.010 (4)
C28	0.044 (7)	0.039 (6)	0.034 (5)	0.002 (5)	0.010 (5)	0.007 (4)
C29	0.051 (7)	0.040 (6)	0.050 (6)	−0.009 (5)	0.004 (5)	−0.002 (5)
C30	0.069 (9)	0.044 (7)	0.065 (8)	0.007 (6)	0.021 (7)	0.022 (6)
C31	0.081 (10)	0.051 (7)	0.044 (7)	0.003 (6)	0.020 (6)	0.024 (5)
C32	0.078 (9)	0.060 (8)	0.034 (6)	0.011 (7)	0.012 (6)	0.011 (5)
O33	0.084 (6)	0.048 (4)	0.028 (4)	−0.016 (4)	0.003 (4)	−0.001 (3)

### Geometric parameters (Å, º)

**Table:**

Cu1—O17	1.864 (7)	C15—H15	0.9300
Cu1—O33	1.869 (7)	C16—H16	0.9300
Cu1—N18	1.983 (7)	N18—C26	1.347 (11)
Cu1—N2	2.004 (7)	N18—C19	1.412 (11)
N2—C10	1.326 (11)	C19—C24	1.392 (12)
N2—C3	1.385 (11)	C19—C20	1.393 (13)
C3—C4	1.383 (13)	C20—C21	1.359 (14)
C3—C8	1.401 (12)	C20—H20	0.9300
C4—C5	1.359 (14)	C21—C22	1.383 (15)
C4—H4	0.9300	C21—H21	0.9300
C5—C6	1.388 (14)	C22—C23	1.377 (16)
C5—H5	0.9300	C22—H22	0.9300
C6—C7	1.372 (15)	C23—C24	1.397 (14)
C6—H6	0.9300	C23—H23	0.9300
C7—C8	1.395 (14)	C24—S25	1.706 (11)
C7—H7	0.9300	S25—C26	1.738 (9)
C8—S9	1.723 (10)	C26—C27	1.404 (13)
S9—C10	1.743 (10)	C27—C32	1.403 (12)
C10—C11	1.413 (13)	C27—C28	1.434 (13)
C11—C16	1.409 (13)	C28—O33	1.301 (10)
C11—C12	1.425 (13)	C28—C29	1.401 (13)
C12—O17	1.316 (10)	C29—C30	1.383 (14)
C12—C13	1.402 (13)	C29—H29	0.9300
C13—C14	1.369 (13)	C30—C31	1.363 (15)
C13—H13	0.9300	C30—H30	0.9300
C14—C15	1.370 (15)	C31—C32	1.365 (15)
C14—H14	0.9300	C31—H31	0.9300
C15—C16	1.355 (15)	C32—H32	0.9300
			
O17—Cu1—O33	147.0 (3)	C15—C16—H16	118.4
O17—Cu1—N18	95.7 (3)	C11—C16—H16	118.4
O33—Cu1—N18	92.7 (3)	C12—O17—Cu1	130.5 (6)
O17—Cu1—N2	93.1 (3)	C26—N18—C19	111.3 (7)
O33—Cu1—N2	96.2 (3)	C26—N18—Cu1	122.7 (6)
N18—Cu1—N2	148.4 (3)	C19—N18—Cu1	125.9 (6)
C10—N2—C3	113.4 (8)	C24—C19—C20	120.9 (9)
C10—N2—Cu1	122.1 (6)	C24—C19—N18	114.1 (8)
C3—N2—Cu1	124.2 (6)	C20—C19—N18	125.0 (8)
C4—C3—N2	128.5 (8)	C21—C20—C19	118.4 (10)
C4—C3—C8	118.3 (9)	C21—C20—H20	120.8
N2—C3—C8	113.3 (8)	C19—C20—H20	120.8
C5—C4—C3	121.1 (9)	C20—C21—C22	121.6 (11)
C5—C4—H4	119.5	C20—C21—H21	119.2
C3—C4—H4	119.5	C22—C21—H21	119.2
C4—C5—C6	120.0 (10)	C23—C22—C21	120.8 (11)
C4—C5—H5	120.0	C23—C22—H22	119.6
C6—C5—H5	120.0	C21—C22—H22	119.6
C7—C6—C5	121.4 (10)	C22—C23—C24	118.6 (10)
C7—C6—H6	119.3	C22—C23—H23	120.7
C5—C6—H6	119.3	C24—C23—H23	120.7
C6—C7—C8	118.0 (10)	C19—C24—C23	119.7 (10)
C6—C7—H7	121.0	C19—C24—S25	110.2 (7)
C8—C7—H7	121.0	C23—C24—S25	130.0 (8)
C7—C8—C3	121.3 (9)	C24—S25—C26	91.7 (4)
C7—C8—S9	128.5 (8)	N18—C26—C27	126.6 (8)
C3—C8—S9	110.1 (7)	N18—C26—S25	112.7 (7)
C8—S9—C10	90.7 (5)	C27—C26—S25	120.8 (7)
N2—C10—C11	127.5 (9)	C32—C27—C26	119.2 (9)
N2—C10—S9	112.6 (7)	C32—C27—C28	117.3 (9)
C11—C10—S9	119.9 (7)	C26—C27—C28	123.5 (8)
C16—C11—C10	120.3 (9)	O33—C28—C29	118.5 (9)
C16—C11—C12	116.6 (10)	O33—C28—C27	122.2 (9)
C10—C11—C12	123.2 (8)	C29—C28—C27	119.3 (8)
O17—C12—C13	118.3 (9)	C30—C29—C28	120.0 (10)
O17—C12—C11	122.9 (9)	C30—C29—H29	120.0
C13—C12—C11	118.8 (9)	C28—C29—H29	120.0
C14—C13—C12	121.4 (10)	C31—C30—C29	120.9 (11)
C14—C13—H13	119.3	C31—C30—H30	119.5
C12—C13—H13	119.3	C29—C30—H30	119.5
C13—C14—C15	120.3 (10)	C30—C31—C32	120.3 (10)
C13—C14—H14	119.8	C30—C31—H31	119.9
C15—C14—H14	119.8	C32—C31—H31	119.9
C16—C15—C14	119.6 (10)	C31—C32—C27	122.0 (10)
C16—C15—H15	120.2	C31—C32—H32	119.0
C14—C15—H15	120.2	C27—C32—H32	119.0
C15—C16—C11	123.2 (11)	C28—O33—Cu1	131.1 (6)

### Hydrogen-bond geometry (Å, º)

**Table:**

*D*—H···*A*	*D*—H	H···*A*	*D*···*A*	*D*—H···*A*
C4—H4···O33	0.93	2.41	2.997 (12)	121
C7—H7···O17^i^	0.93	2.59	3.305 (13)	134
C20—H20···O17	0.93	2.42	3.000 (13)	121
C23—H23···O33^ii^	0.93	2.61	3.303 (13)	132

Symmetry codes: (i) *x*+1/2, −*y*+1/2, *z*−1/2; (ii) *x*+1/2, −*y*+1/2, *z*+1/2.

## References

[bb1] Balashova, T. V., Pushkarev, A. P., Ilichev, V. A., Lopatin, M. A., Katkova, M. A., Baranov, E. V., Fukin, G. K. & Bochkarev, M. N. (2013). *Polyhedron*, **50**, 112–120.

[bb2] Booysen, I. N., Gerber, T. I. A. & Mayer, P. (2010). *Inorg. Chim. Acta*, **363**, 1292–1296.

[bb3] Bruker (2002). *SADABS*, *SAINT* and *SMART*. Bruker AXS Inc., Madison, Wisconsin, USA.

[bb4] Farrugia, L. J. (2012). *J. Appl. Cryst.* **45**, 849–854.

[bb5] Henary, M. M. & Fahrni, C. J. (2002). *J. Phys. Chem. A*, **106**, 5210–5220.

[bb6] Katkova, M. A., Pushkarev, A. P., Balashova, T. V., Konev, A. N., Fukin, G. K., Ketkov, S. Y. & Bochkarev, M. N. (2011). *J. Mater. Chem.* **21**, 16611–16620.

[bb7] Liu, Y., Li, M., Zhao, Q., Wu, H., Huang, K. & Li, F. (2011). *Inorg. Chem.* **50**, 5969–5977. 10.1021/ic102481x 21639129

[bb8] López-Banet, L., Santana, M. D., Piernas, M. J., García, G., Cerezo, J., Requena, A., Zúñiga, J., Pérez, J. & García, L. (2014). *Inorg. Chem.* **53**, 5502–5514. 10.1021/ic5001655 24848344

[bb9] Sheldrick, G. M. (2008). *Acta Cryst.* A**64**, 112–122. 10.1107/S0108767307043930 18156677

[bb10] Sheldrick, G. M. (2015). *Acta Cryst.* C**71**, 3–8.

[bb11] Wang, K., Huang, L., Gao, L., Huang, C. & Jin, L. (2002). *Solid State Commun.* **122**, 233–236.

[bb12] Westrip, S. P. (2010). *J. Appl. Cryst.* **43**, 920–925.

[bb13] Yu, G., Yin, S., Liu, Y., Shuai, Z. & Zhu, D. (2003). *J. Am. Chem. Soc.* **125**, 14816–14824. 10.1021/ja0371505 14640657

