# Supplementary figures and images for: Crystal structure of bis­[2-(benzo­thia­zol-2-yl)phenolato-κ2 N,O]copper(II)
Source: Acta Crystallogr E Crystallogr Commun. 2015 Aug 22;71(Pt 9):m173–4. doi: 10.1107/S2056989015015303 (PMC4555420; doi:10.1107/S2056989015015303)

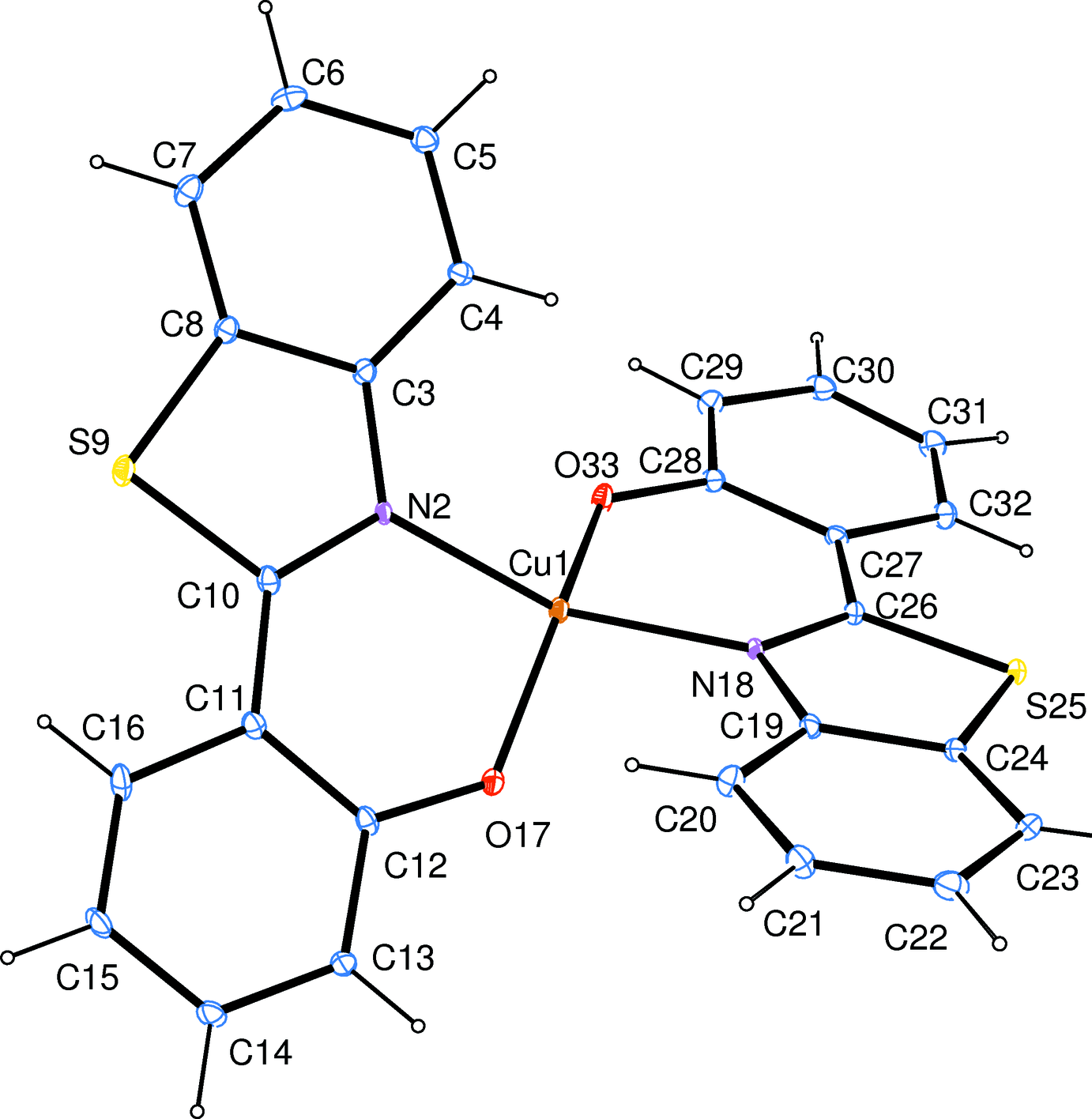

Supplement: Supplementary file 3 [file e-71-0m173-fig1.tif]

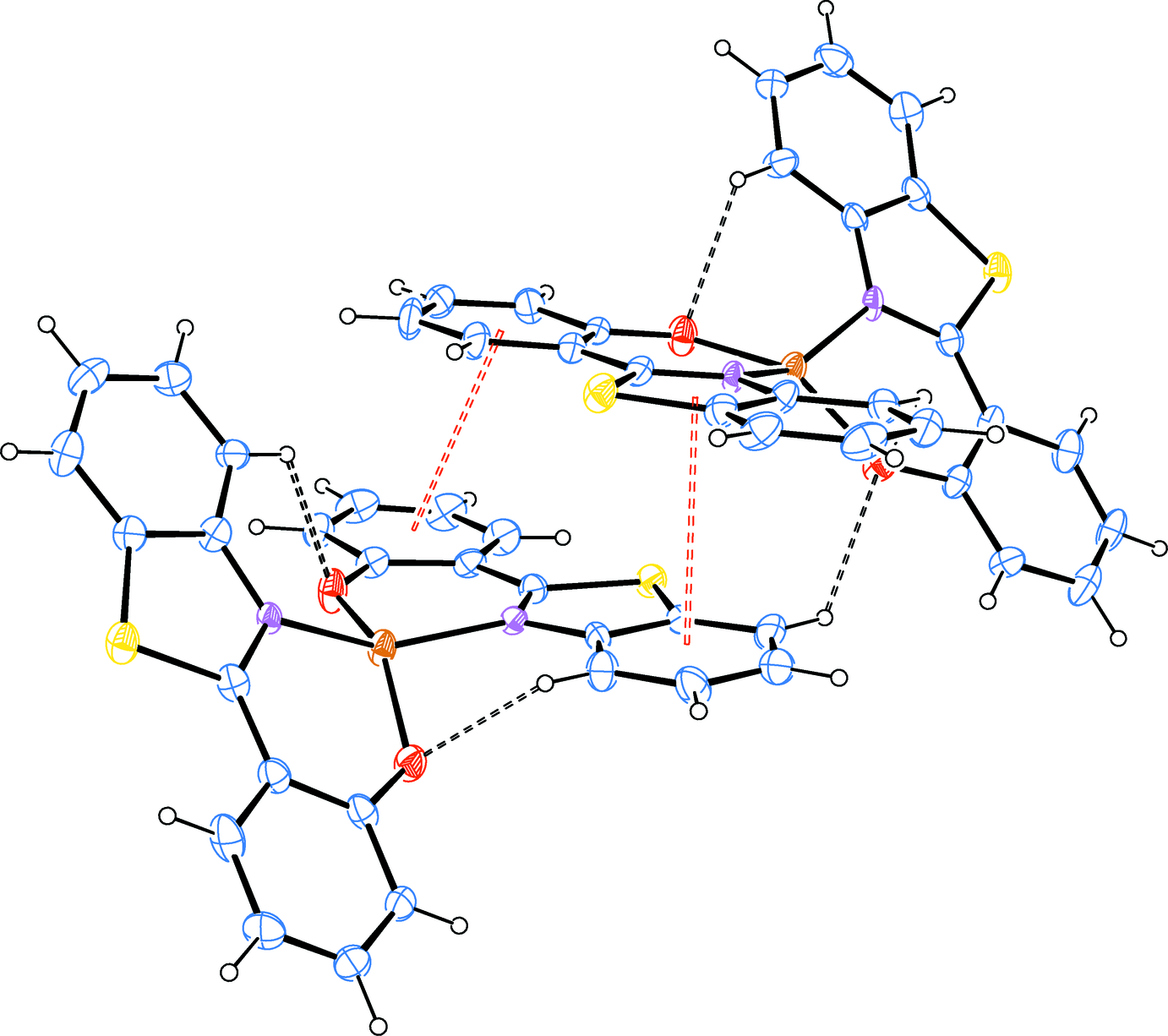

Supplement: Supplementary file 4 [file e-71-0m173-fig2.tif]

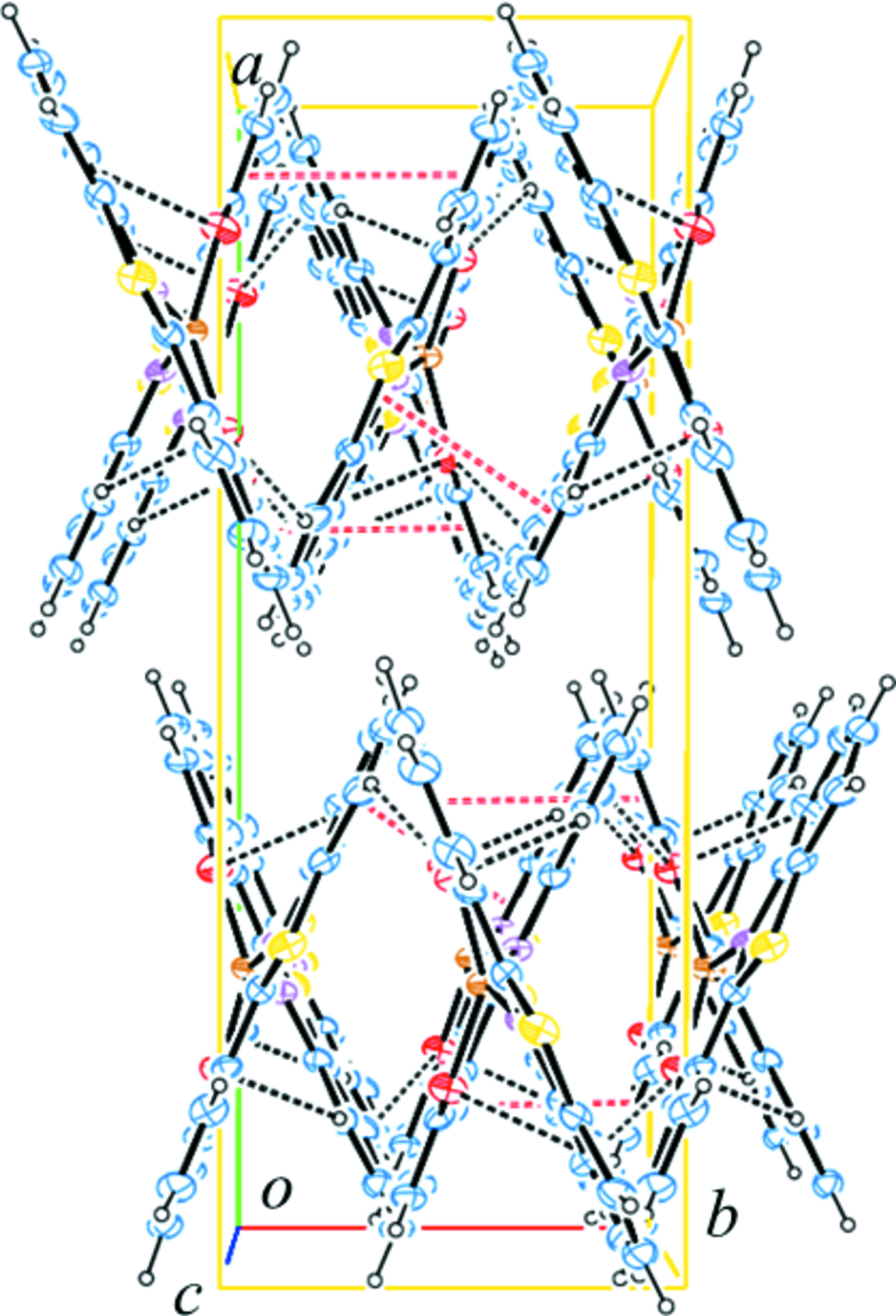

Supplement: Supplementary file 5 [file e-71-0m173-fig3.tif]
